# Supplementary material for: Machine Learning Accelerates Crystallization for Structure Determination
Source: Angew Chem Int Ed Engl. 2026 May 4;65(25):e1218503. doi: 10.1002/anie.1218503 (PMC13266958; doi:10.1002/anie.1218503)

checkCIF/PLATON report

Structure factors have been supplied for datablock(s) lcz034

THIS REPORT IS FOR GUIDANCE ONLY. IF USED AS PART OF A REVIEW PROCEDURE FOR PUBLICATION, IT SHOULD NOT REPLACE THE EXPERTISE OF AN EXPERIENCED CRYSTALLOGRAPHIC REFEREE.

No syntax errors found. CIF dictionary Interpreting this report

Datablock: lcz034

Bond precision:	C-C = 0.0181 A		Wavelength=1.54184
Cell:	a=10.8243(2)	b=17.4480(2)	c=17.0198(3)
	alpha=90	beta=94.186(2)	gamma=90
Temperature:	100 K		

	Calculated	Reported
Volume	3205.83(9)	3205.82(9)
Space group	P 21/c	P 1 21/c 1
Hall group	-P 2ybc	-P 2ybc
Moiety formula	C25 H13 Ag3 F18 N6 O [+ solvent]	C15 H3 Ag3 F18 N6, C10 H10 O
Sum formula	C25 H13 Ag3 F18 N6 O [+ solvent]	C25 H13 Ag3 F18 N6 O
Mr	1079.03	1079.02
Dx,g cm-3	2.236	2.236
Z	4	4
Mu (mm-1)	15.866	15.866
F000	2064.0	2064.0
F000’	2074.05	
h,k,lmax	13,22,21	13,21,21
Nref	6852	6542
Tmin,Tmax	0.109,0.108	0.439,1.000
Tmin’	0.009	

```
Correction method= # Reported T Limits: Tmin=0.439 Tmax=1.000
AbsCorr = MULTI-SCAN

Data completeness= 0.955          Theta(max)= 77.843

                                                      wR2(reflections)=
R(reflections)= 0.0944( 5135)                         0.2206( 6542)

S = 1.065                Npar= 461
```

The following ALERTS were generated. Each ALERT has the format
test-name_ALERT_alert-type_alert-level.
Click on the hyperlinks for more details of the test.

Alert level C

```
PLAT213_ALERT_2_C Atom F007            has ADP max/min Ratio .....        3.3 prolat
PLAT213_ALERT_2_C Atom F00C            has ADP max/min Ratio .....        4.0 prolat
PLAT213_ALERT_2_C Atom F00G            has ADP max/min Ratio .....        3.3 prolat
PLAT213_ALERT_2_C Atom F00M            has ADP max/min Ratio .....        3.4 prolat
PLAT213_ALERT_2_C Atom F00Q            has ADP max/min Ratio .....        3.6 prolat
PLAT234_ALERT_4_C Large Hirshfeld Difference F009     --C019     .       0.18 Ang.
PLAT234_ALERT_4_C Large Hirshfeld Difference C012     --C01F     .       0.20 Ang.
PLAT342_ALERT_3_C Low Bond Precision on  C-C Bonds ..............    0.01809 Ang.
PLAT906_ALERT_3_C Large K Value in the Analysis of Variance ......     17.878 Check
PLAT906_ALERT_3_C Large K Value in the Analysis of Variance ......      4.117 Check
PLAT906_ALERT_3_C Large K Value in the Analysis of Variance ......      2.187 Check
PLAT911_ALERT_3_C Missing FCF Refl Between Thmin & STh/L=    0.600         47 Report
              12  0  0,  12  1  1,  12  7  1,  -5  0  2,  12  0  2,  12  4  2,
              12  5  2,  12  6  2,  11  9  2,  -3 20  2,  11  2  3,   1  3  3,
              11  7  3,  11  8  3,  10  9  3,  -5 17  3,  10  0  4,  11  5  4,
              11  7  4,   9 11  4,  10  3  5,   1 17  5,   8  0  6,   8  1  6,
               9  3  6,   4  4  6,  11  1  7,  11  4  7,  -6 13  7,  -8  0  8,
                  (   17 More Missing: see the .ckf listing file)
PLAT971_ALERT_2_C Check Calcd Resid. Dens.  1.39Ang From F00J          1.62 eA-3
PLAT971_ALERT_2_C Check Calcd Resid. Dens.  0.93Ang From Ag02          1.58 eA-3
PLAT971_ALERT_2_C Check Calcd Resid. Dens.  0.93Ang From C00U          1.56 eA-3
PLAT971_ALERT_2_C Check Calcd Resid. Dens.  0.87Ang From Ag01          1.54 eA-3
PLAT971_ALERT_2_C Check Calcd Resid. Dens.  0.76Ang From O00N          1.52 eA-3
PLAT972_ALERT_2_C Check Calcd Resid. Dens.  1.01Ang From Ag02         -2.45 eA-3
PLAT972_ALERT_2_C Check Calcd Resid. Dens.  0.79Ang From Ag01         -2.34 eA-3
PLAT972_ALERT_2_C Check Calcd Resid. Dens.  0.78Ang From Ag03         -1.91 eA-3
PLAT972_ALERT_2_C Check Calcd Resid. Dens.  0.87Ang From Ag02         -1.79 eA-3
PLAT972_ALERT_2_C Check Calcd Resid. Dens.  0.30Ang From F00R         -1.72 eA-3
PLAT972_ALERT_2_C Check Calcd Resid. Dens.  0.85Ang From Ag03         -1.67 eA-3
PLAT972_ALERT_2_C Check Calcd Resid. Dens.  1.11Ang From N00D         -1.60 eA-3
```

Alert level G

```
PLAT003_ALERT_2_G Number of Uiso or U(i,j)  Restrained non-H-Atoms         29 Report
PLAT042_ALERT_1_G Calc. and Reported MoietyFormula Strings  Differ     Please Check
              Calc: C25 H13 Ag3 F18 N6 O
              Rep.: C15 H3 Ag3 F18 N6, C10 H10 O
PLAT083_ALERT_2_G SHELXL Second Parameter in WGHT  Unusually Large     211.15 Why ?
```

```
PLAT171_ALERT_4_G The CIF-Embedded .res File Contains EADP Records          1 Report
PLAT177_ALERT_4_G The CIF-Embedded .res File Contains DELU Records          6 Report
PLAT178_ALERT_4_G The CIF-Embedded .res File Contains SIMU Records          1 Report
PLAT186_ALERT_4_G The CIF-Embedded .res File Contains ISOR Records          5 Report
PLAT242_ALERT_2_G Low    'MainMol' Ueq as Compared to Neighbors of       C00U Check
PLAT242_ALERT_2_G Low    'MainMol' Ueq as Compared to Neighbors of       C017 Check
PLAT242_ALERT_2_G Low    'MainMol' Ueq as Compared to Neighbors of       C018 Check
PLAT242_ALERT_2_G Low    'MainMol' Ueq as Compared to Neighbors of       C019 Check
PLAT242_ALERT_2_G Low    'MainMol' Ueq as Compared to Neighbors of       C01B Check
PLAT434_ALERT_2_G Short Inter HL..HL Contact F00O     ..F00O     .       2.79 Ang.
                                              1-x,2-y,1-z  =      3_676 Check
PLAT720_ALERT_4_G Number of Unusual/Non-Standard Labels ..........         66 Note
              Ag01    Ag02    Ag03    F004    F005    F006    F007    F008
              F009    N00A    F00B    F00C    N00D    F00E    N00F    F00G
              F00H    N00I    F00J    F00K    N00L    F00M    O00N    F00O
              N00P    F00Q    F00R    F00S    C00T    H00T    C00U    C00V
              H00A    H00B    H00C    C00W    C00X    C00Y    C00Z    H00Z
              C011    C013    H013    C016    H016    C012    C010    H010
              C01D    H01D    C01G    H01G    C014    C015    C017    C018
              C019    C01A    C01B    C01C    H01C    C01E    H01E    C01F
              H01F    C01H
PLAT860_ALERT_3_G Number of Least-Squares Restraints ............         130 Note
PLAT868_ALERT_4_G ALERTS Due to the Use of _smtbx_masks Suppressed          ! Info
PLAT883_ALERT_1_G Absent Datum for _atom_sites_solution_primary ..     Please Do !
PLAT912_ALERT_4_G Missing # of FCF Reflections Above STh/L=  0.600        257 Note
PLAT933_ALERT_2_G Number of HKL-OMIT Records in Embedded .res File         14 Note
               -6 13  7,   5 17  9,   8  9 14,   9 11  4,  10  2  8,  10  6  8,
               10  7  8,  11  1  7,  11  4  7,  11  5  4,  12  4  2,  13  1  4,
               13  2  4,  13  3  3,
PLAT941_ALERT_3_G Average HKL Measurement Multiplicity ..........         2.4 Low
PLAT969_ALERT_5_G The 'Henn et al.' R-Factor-gap value ..........       3.580 Note
              Predicted wR2: Based on SigI**2  6.16 or SHELX Weight 20.72
PLAT978_ALERT_2_G Number C-C Bonds with Positive Residual Density.          0 Info
```

```
   0 ALERT level A = Most likely a serious problem - resolve or explain
   0 ALERT level B = A potentially serious problem, consider carefully
  24 ALERT level C = Check. Ensure it is not caused by an omission or oversight
  22 ALERT level G = General information/check it is not something unexpected

   2 ALERT type 1 CIF construction/syntax error, inconsistent or missing data
  27 ALERT type 2 Indicator that the structure model may be wrong or deficient
   7 ALERT type 3 Indicator that the structure quality may be low
   9 ALERT type 4 Improvement, methodology, query or suggestion
   1 ALERT type 5 Informative message, check
```

Validation response form

Please find below a validation response form (VRF) that can be filled in and pasted into your CIF.

```
# start Validation Reply Form
_vrf_PLAT213_lcz034
;
PROBLEM: Atom F007            has ADP max/min Ratio .....        3.3 prolat
RESPONSE: ...
;
```

```
_vrf_PLAT234_lcz034
;
PROBLEM: Large Hirshfeld Difference F009     --C019     .       0.18 Ang.
RESPONSE: ...
;
_vrf_PLAT342_lcz034
;
PROBLEM: Low Bond Precision on  C-C Bonds ..............    0.01809 Ang.
RESPONSE: ...
;
_vrf_PLAT906_lcz034
;
PROBLEM: Large K Value in the Analysis of Variance ......     17.878 Check
RESPONSE: ...
;
_vrf_PLAT911_lcz034
;
PROBLEM: Missing FCF Refl Between Thmin & STh/L=    0.600         47 Report
RESPONSE: ...
;
_vrf_PLAT971_lcz034
;
PROBLEM: Check Calcd Resid. Dens.  1.39Ang From F00J            1.62 eA-3
RESPONSE: ...
;
_vrf_PLAT972_lcz034
;
PROBLEM: Check Calcd Resid. Dens.  1.01Ang From Ag02           -2.45 eA-3
RESPONSE: ...
;
# end Validation Reply Form
```

It is advisable to attempt to resolve as many as possible of the alerts in all categories. Often the minor alerts point to easily fixed oversights, errors and omissions in your CIF or refinement strategy, so attention to these fine details can be worthwhile. It is up to the individual to critically assess their own results and, if necessary, seek expert advice.

PLATON version of 17/09/2025; check.def file version of 17/09/2025

---

duplicate check

No duplication found

Datablock lcz034 - ellipsoid plot

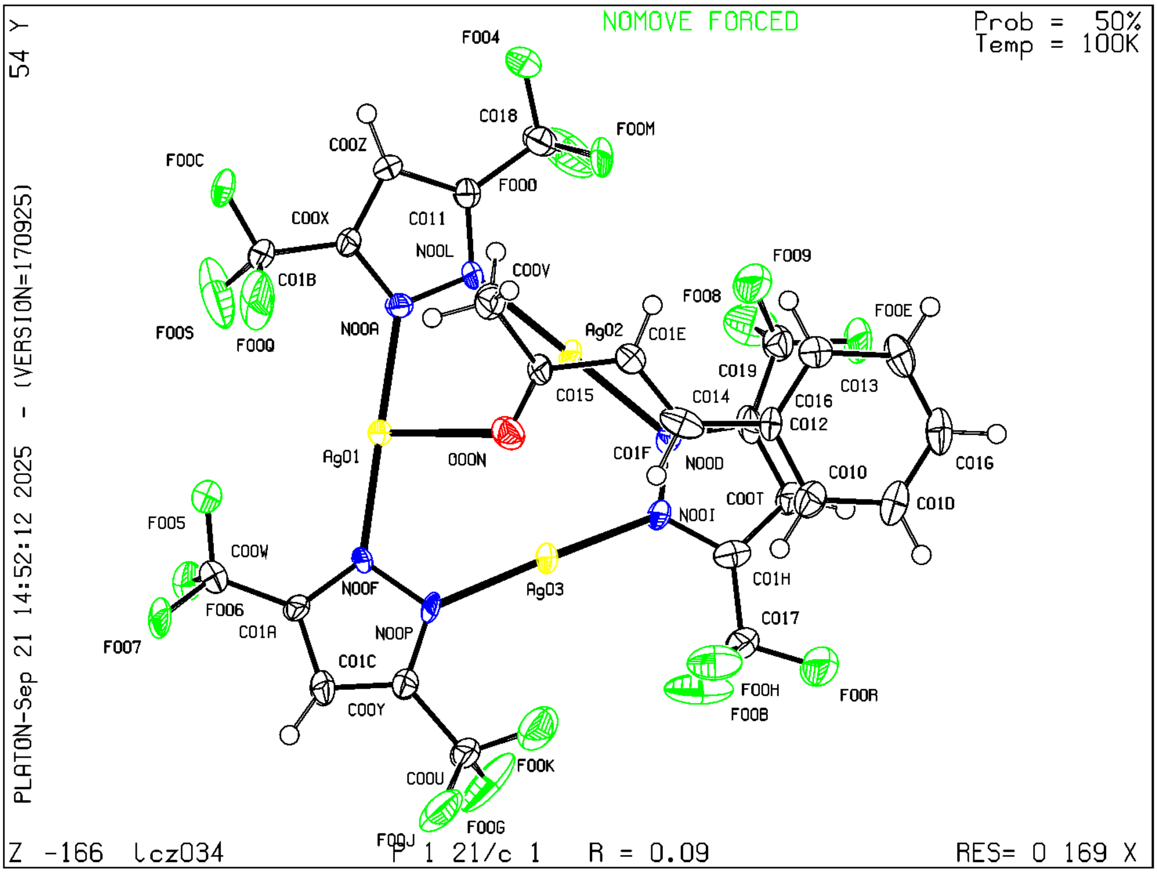

Supplement: Supplementary file 2 — Supporting File 2: anie72462‐sup‐0002‐DataFile.zip. [file ANIE-65-e1218503-s002.zip › supplementary_materials_crystallographic_data submit.zip!/cif_and_checkcif(1-114)/Ag3Pz3_34/Ag3Pz3_34_checkcif.pdf]
